# Curcumin as a Holistic Treatment for Tau Pathology

**DOI:** 10.3389/fphar.2022.903119

**Published:** 2022-05-19

**Authors:** Lovesha Sivanantharajah, Amritpal Mudher

**Affiliations:** ^1^ School of Natural Sciences, Bangor University, Bangor Gwynedd, United Kingdom; ^2^ Faculty of Natural and Environmental Sciences, University of Southampton, Southampton, United Kingdom

**Keywords:** turmeric (Curcuma longa L), curcumin, Alzheheimer’s disease, tauopathy, animal models, holistic medicine

## Abstract

Global forecasts for prevalence of Alzheimer’s Disease (AD) estimate that 152.8 million people will have dementia in 2050, a sharp rise from 57.4 million in 2019 (GBD 2019). This rise can be attributable to increases in population growth and aging, but in the absence of disease-modifying therapies it poses a huge societal challenge that must be addressed urgently. One way to combat this challenge is to explore the utility of holistic treatments that may protect against AD, including traditional herbs, spices and other nutraceuticals that are pharmacologically safe, inexpensive and readily available. In this light, the spice turmeric, and its active ingredient curcumin, has been investigated as a potential holistic treatment for AD over the past 2 decades; however, promising results with animal studies have not translated to success in clinical trials. One issue is that most animal models examining the effects of curcumin and curcumin derivatives in AD have been done with a focus at ameliorating amyloid pathology. Due to the limited success of Amyloid-β-based drugs in recent clinical trials, tau-focused therapeutics provide a promising alternative. In this article, we aim to provide a clearer picture of what is currently known about the effectiveness of curcumin and curcumin derivatives to ameliorate tau pathology. Tau focused studies may help inform more successful clinical studies by placing greater emphasis on the development and optimised delivery of curcumin derivatives that more effectively target tau pathology.

## Introduction

Alzheimer’s disease (AD) is the most common form of dementia, characterised clinically by progressive cognitive decline, and neuropathologically by amyloid plaques and neurofibrillary tau tangles (NFT). Global forecasts for prevalence of AD estimate that 152.8 million people will have dementia in 2050, a sharp rise from 57.4 million in 2019 (GBD 2019). Although this rise can be attributable to increases in population growth and aging, in the absence of disease-modifying therapies it poses a huge societal challenge we must urgently address. One approach is to implement public health measures that scale up modifiable lifestyle factors known to influence risk of AD (GBD 2019) (Livingston et al., 2020). Another is to explore the utility of holistic treatments that may protect against AD, including traditional herbs, spices and other nutraceuticals that are pharmacologically safe, inexpensive and readily available. The case for repurposing such nutraceuticals for dementia treatment first arose from the observation that the prevalence of dementia in countries like India, where such spices are regularly consumed, is remarkably low when compared to high income countries where these spices are not common (Kannappan et al., 2011). Although other factors, including population ageing differences, have since been put forward to explain at least some of this differential dementia prevalence, there have been significant efforts to assess the disease-modifying potential of nutraceuticals in cellular and animal models of AD. Such research reveals that common Indian herbs and spices such as turmeric, garlic, ginger, black pepper, coriander and others, contain phytochemicals that display a range of neuroprotective properties (Kannappan et al., 2011). They have been shown to be antioxidant, anti-inflammatory, anti-amyloidogenic and also to upregulate protective cellular responses like heat shock responses (Forouzanfar et al., 2019). As misfolding and aggregation of tau and amyloid proteins, oxidative stress and aberrant inflammation are all implicated in AD, it is not surprising that these spice-derived nutraceuticals have shown some disease-modifying potential in AD models. This review summarises the research done to investigate the protective effects of the spice, curcumin, against tau pathology in AD.

## Curcumin and Alzheimer’s Disease

The medicinal properties of the nutraceutical, turmeric, have been known for centuries, prompting its widespread use in holistic traditional medicines in India and China. It is derived from the rhizome of *Curcuma longa*, a member of the ginger family indigenous to South and Southeast Asia. The active ingredient in turmeric is the phytochemical, curcumin ((1E, 6E)-1, 7-bis (4-hydroxy-3-methoxyphenyl)-1, 6-heptadiene-3, 5-dione). As a key ingredient in Asian cooking, turmeric was first considered for prevention or treatment of AD because of reports that prevalence of AD in India was significantly lower than in the West where this spice is not routinely consumed (Kannappan et al., 2011). Although more recent studies have begun to show that several other lifestyle related factors modulate AD risk, and that prevalence may be increasing in Asian countries and decreasing in the West (Livingston et al., 2020), these original observations prompted research into the disease-modifying potential of turmeric and its active ingredient, curcumin. Further, as evidence mounts for the importance of diet on wellbeing and disease prevention, curcumin becomes a more attractive medicinal agent providing a cost-effective, easily accessible solution to promote resilience to dementia. This will have even greater significance for developing countries.

Curcumin has a wide variety of neuroprotective properties: it inhibits nuclear factor kB-mediated transcription of inflammatory cytokines (Singh and Aggarwal 1995), it behaves as an anti-oxidant by scavenging reactive oxygen species (ROS) (Scapagnini et al., 2010)and neutralising NO-based free radicals (Sreejayan and Rao 1997), it is capable of disrupting amyloids displaying anti-amyloidogenic and anti-fibrillogenic behaviour (Garcia-Alloza et al., 2007), and interacts with homeostatic pathways such as the unfolded protein response (UPR) and chaperones like Hsp70 to facilitate correct protein folding (Mukherjee et al., 2021). Curcumin has also been shown to play a protective role in aging and age-related disorders including atherosclerosis, diabetes and cancer (Olszanecki et al., 2005; He et al., 2015). One striking observation is the ability of dietary curcumin to extend life span in fly, nematode and mouse models (Suckow and Suckow 2006; Kitani et al., 2007; Lee et al., 2010; Liao et al., 2011). Therefore, it is not surprising that curcumin and its derivatives have been found to suppress phenotypes in multiple models of neurodegenerative disease in which aging is a substantive risk factor, including Huntington’s Disease (Chongtham and Agrawal 2016; Labanca et al., 2021), Parkinson’s Disease (Chetty et al., 2021; Nebrisi 2021)), and AD (Reddy et al., 2018; Chainoglou and Hadjipavlou-Litina 2020). The observation that curcumin treatment leads to suppression of phenotypes caused by a wide range of conditions, from aging-related to several different misfolded proteins characterising distinct proteinopathies, suggests that its disease-modifying actions may be affected through key overlapping mechanisms such as oxidative stress and neuroinflammation.

## The Status Quo

In the absence of a cure for AD, there are four drugs in general use for the symptomatic treatment of AD. Three of these drugs—donepezil, galantamine and rivastigmine—are acetylcholinesterase (AChE) inhibitors, developed on the cholinergic hypothesis which proposed AD was due to preferential loss of cholinergic neurons (Bartus 2000). The fourth drug, memantine, is a N-methyl-D-aspartate (NMDA) receptor antagonist, which suppresses the neuronal excitotoxicity noted in AD caused by excess glutamate binding of NMDA receptors (Reisberg et al., 2003). More recently, the Federal Drug Administration (USA) granted accelerated approval to the drug, aducanumab, which works by reducing amyloid deposits in the brain that may slow the progression of AD; however, it’s effectiveness in halting the progression of cognitive decline or dementia has yet to be clinically demonstrated.

Tau-directed treatments have also been investigated, and over the years a number of different tau-centric agents have entered clinical trials with limited success. (Panza et al., 2016). These include kinase inhibitors (Lovestone et al., 2015; Maqbool et al., 2016), phosphatase stimulators (Parekh et al., 2013), aggregation inhibitors like methylene blue (Wischik et al., 1996), tau vaccines (d'Abramo et al., 2015; Sankaranarayanan et al., 2015), and microtubule stabilising agents (Brunden et al., 2011; Quraishe et al., 2016). However, in the absence of approved tau-modifying treatments or in fact any disease modifying treatments, current medical treatments arguably provide small benefits at the cost of side effects and lack general accessibility. It is here that holistic medicines, such as curcumin, can play a key role.

To date, numerous animal studies have been conducted, mainly in rodent models, with promising results for the effectiveness of curcumin for therapeutic purposes (see comprehensive review of animal and clinical studies in Voulgaropoulou et al. (2019). Unfortunately, this has not translated to similar success in human clinical trials where study results are inconsistent, with some studies reporting a beneficial effect of curcumin on cognitive function (Cox et al., 2015; Rainey-Smith et al., 2016; Small et al., 2018) and others reporting none (Baum et al., 2008; Ringman et al., 2012). Since so few clinical studies have actually been done, this may not be surprising. However, many factors may contribute to the observed discrepancy in translation with the first being issues with curcumin supplementation, which include low bioavailability of the compound, rapid metabolism in gut, and low penetration across the blood brain barrier (Anand et al., 2007). There are a number of ways in which these issues have been, and are currently being, addressed with the development of curcumin derivatives with greater bioavailability and optimisation of delivery methods, including isomerisation, liposomes, micelles, phospholipids, nanotechnology, vaporising and intravenous injection.

Coupled with the above issue is that much of the work examining the effects of curcumin in AD in animal models has been done with a focus at ameliorating amyloid pathology. However, the limited success of Amyloid-beta (Aβ)-based drugs in clinical trials suggests a shift in focus is needed. This is where tau-targeted therapeutics provide a promising alternative. Currently, few studies have directly examined the effectiveness of curcumin and curcumin derivatives in tauopathy models or to ameliorate tau pathology in animal and clinical studies. Here we provide a snapshot of this field with a view that tau-focused animal studies may help inform more successful clinical studies by emphasising development of curcumin derivatives and delivery mechanisms that more effectively target tau pathology.

## Animal Models Examining Curcumin and Tau Pathology

Tauopathies are a class of over 20 degenerative disorders, including AD, marked by neuronal aggregation of abnormally phosphorylated forms of the protein tau, a microtubule associated protein (MAPT) essential for microtubule assembly and stability ((Grundke-Iqbal et al., 1986; Williams 2006; Spillantini and Goedert 2013). In pathological scenarios, there is evidence of both toxic loss of function (LOF) as well as gain of toxic functions (GOF) by pathogenic tau. Hyperphosphorylated tau detaches from microtubules and causes their destabilisation, consequently leading to disruption of cytoskeletal integrity, axonal transport and synaptic transmission (Mudher et al., 2004; Chee et al., 2005; Cowan et al., 2010; Hoover et al., 2010). Further, these abnormally hyperphosphorylated tau proteins aggregate to form higher order structures from soluble oligomers to paired helical filaments (PHFs) to insoluble neurofibrillary tangles (NFTs) ((Spillantini and Goedert 2013; von Bergen et al., 2000). While PHFs have historically been thought of as the main pathological species in tauopathies, more recent studies have suggested that it is their precursors, soluble tau oligomers, that are the toxic species spurring disease pathogenesis and progression through a variety of mechanisms including oxidative stress (Stamer et al., 2002; Cowan et al., 2021), mitochondrial dysfunction and transport (Ittner et al., 2008), nuclear dysfunction (Andorfer et al., 2005; Khurana et al., 2012), prion-like conversion and propagation of pathology (Holmes et al., 2014), inflammation (Nagele et al., 2004; Ismail et al., 2020), and induction of ER stress (Hoozemans et al., 2009) (Götz et al., 2019). Thus, finding ways to target toxic tau species is one solution for developing disease-modifying treatments that ameliorate or prevent these LOF and GOF pathogenic effects.

**FIGURE 1:**
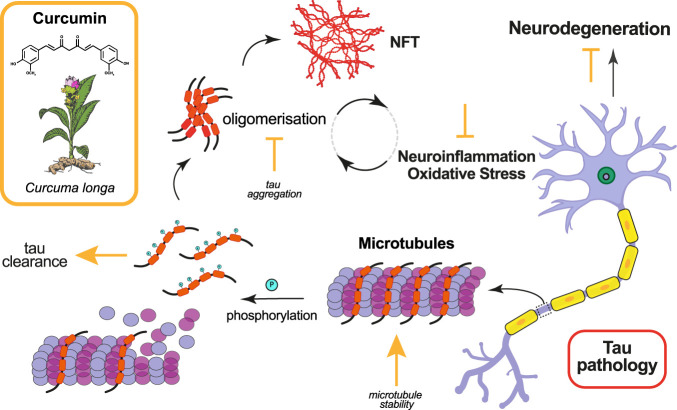
Effects of curcumin in ameliorating tau pathology. Curcumin and its derivatives have been shown to ameliorate tau pathology in animal models by targeting various molecular mechanisms. The mode of action of curcumin, whether promotive (arrow) or inhibitive (falsum), is indicated in yellow next to the process it modifies.

While the vast majority of animal studies with curcumin have been done in models of amyloid pathology, there are a handful that have examined the effects of curcumin in ameliorating tau pathology (Table 1, Figure 1). Using a nematode model of tauopathy, Miyasaka et al. (Miyasaka et al., 2016) expressed wild-type human tau isoform 0N4R (hTau0N4R) and hTau with the R406W (hTauR406W) mutation associated with frontotemporal lobe dementia (FTD). Worms were grown for 3 days on growth medium plates with or without curcumin (at concentrations of 0, 3, 30 uM) before collection of behavioural data. They found that htau-expressing worms grown on 30 uM of curcumin showed significant improvement in behavioural abnormalities (e.g., uncoordinated movement and touch sense). In addition, curcumin significantly decreased the number of morphological abnormalities (i.e., kinks and protrusions) noted in neurons expressing hTau. Interestingly, curcumin treatment had no effect on the phosphorylation of tau (AT100, AT8, AT180, pS262, PHF-1). However, further analysis found that curcumin significantly increased the levels of acetylated alpha-tubulin in worms. Since acetylation of alpha-tubulin occurs in association with microtubule stabilization, one possible mechanism for the therapeutic effects of curcumin is that it enhances microtubule stability.

**TABLE 1:** Summary of animal studies examining curcumin and tau pathology.

References	Goal	Model	Study Design	Curcumin Treatment	Behavioural/Cognitive Measurements	Summary Results
Ma et al. (2013)	Study effects of curcumin on tau pathology in a mouse model	Transgenic mouse model expressing human wild-type Tau (hTau) on a mouse-Tau knockout background	Wildtype versus hTau mice with and without compound for 4 months	500 ppm Longvida in chow	Morris Water Maze (MWM), Y-Maze, Novel Object Recognition task (NORT)	Curcumin reduced the level of soluble tau dimers, reversed the disruption in expression of molecular chaperones (e.g., HSP90, HSP70, HSP72) that is noted in htau-expressing mice. Curcumin treatment improved spatial learning and memory (MWM), when compared to untreated htau mice. Also, curcumin treated htau mice showed normalisation of recognition memory toward wildtype measures (NORT)
Miyasaka et al. (2016)	Establish nematode tauopathy model and test effectiveness of curcumin in reducing tau induced phenotypes	Wildtype hTau0N4R and hTauR406W expressed in *C. elegans*	Worms grown on growth medium plates with or without curcumin for 3 days	0, 3 uM, 30 uM Curcumin in growth medium	Measured uncoordinated movement, Touch sense	Curcumin significantly decreased the number of morphological abnormalities (kinks and protrusions) noted in neurons expressing hTau. hTau expressing worms grown on 30 uM of curcumin showed significant decreases in behavioural abnormalities. Curcumin treatment did not affect the phosphorylation of tau (AT100, AT8, AT180, pS262, PHF-1)
Okuda et al. (2017)	Test the efficiency of their curcumin derivative, PE859, a dual inhibitor of tau and amyloid pathology	Senescence-accelerated mouse prone 8 (SAMP8) model	9-weeks study, SAMP8/TaSlc mice from 2 months of age to 4 months	0, 1 mg/k/day, 3 mg/kg/day PE859, oral administration using gastric tube	Rotarod, MWM, Y-Maze, Grip strength	Saw a reduction in tau and A-beta1-40 aggregates with PE859 treatment. Mice given curcumin treatment did show a significant improvement in cognitive deficits in spatial working memory (Y-Maze). However, no differences noted with other behaviours assayed.
Sundaram et al. (2017)	Examine effects of Longvida^®^ on mouse model of neuroinflammation and neurodegeneration	Transgenic mouse overexpressing p25 (p25Tg)	12-weeks study, Wildtype versus p25Tg mice fed with and without compound	4 g/kg Longvida (0.8 g curcumin/kg) in chow	8-arm radial maze	Saw that curcumin-mediated suppression of neuroinflammation reduced the progression of p25-induced tau/amyloid pathology and ameliorated p25-induced cognitive impairments
Yanagisawa et al. (2018)	Test the effects of their curcumin derivative, Shiga-Y5 (SY5), in a mouse model of tauopathy	Transgenic mouse model rTg4510 expressing a repressible form of human tau with P301L mutation linked with familial frontotemporal dementia	2 month-old male rTg4510 mice and 2 month-old male wild- type mice fed a standard chow diet with or without SY5 for 4 months	500 ppm SY5 in chow	Rotarod, MWM, Y-Maze	Found no significant differences in behavioural performance between rTg4510 mice fed SY5 or a control diet. Further, histological and biochemical analyses found no significant changes in tau accumulation following curcumin treatment

The more common animal model for evaluating curcumin on tau pathology has been the mouse. Ma et al. (Ma et al., 2013) used a transgenic mouse model expressing human wild-type Tau (hTau) on a mouse-Tau knockout background, where synaptic and cognitive deficits and NFTs are present at 11–12 months and neuron loss occurs by 17 months. The mice used in this 4-months study were 15–16 months old at study commencement and were placed on diets without or with 500 ppm Longvida^©^ (Verdure Sciences, Indianapolis, IN) curcumin, solid lipid curcumin particles showing higher bioavailability of free curcumin in the brain compared to other curcumin formulations. This study found that curcumin reduced the level of soluble tau dimers, reversed the disruption noted in htau-expressing mice in expression of the molecular chaperones, HSP90, HSP70, and HSP72, and improved cognitive performance in some behavioural assays. More specifically, they found that curcumin treatment improved spatial learning and memory, as measured using a Morris Water Maze (MWM), when compared to untreated htau mice. Also, curcumin treated htau mice showed normalisation of recognition memory toward wildtype measures using the Novel Object Recognition test (NORT).

Another study also using Longvida^©^ examined abnormal accumulation of NFT’s and Aβ in a p25 transgenic mouse model (Sundaram et al., 2017). The authors found that curcumin treatment significantly reduced p25-mediated tau hyperphosphorylation (AT8) and Aβ1-42 immunostaining in curcumin-treated p25Tg mice compared to their non-treated counterparts. One explanation for this reduction is the suppressed expression of neuroinflammatory cytokines. The authors found that the expression levels of the pro-inflammatory cytokines MIP-1α (macrophage inflammatory protein-1α), TNF-α (tumour necrosis factor-alpha), and IL-1β (Interleukin-1α) were significantly reduced in curcumin treated p25Tg mice. Further, behavioural studies using an 8-arm radial maze showed improved spatial and working memory with curcumin treatment.

Other labs have both developed and tested the effectiveness of curcumin derivatives in mouse models. Yanagisawa et al. (Yanagisawa et al., 2018) examined a novel curcumin derivative synthesised by the group called Shiga-Y5 (SY5), which they had previously found to be effective in inhibiting cognitive impairment and amyloid deposition in an AD mouse model (Yanagisawa et al., 2015). In this study, control and SY5-containing (500 ppm) chow diets were fed for 4 months to rTg4510 mice, a mouse tauopathy model expressing human four-repeat tau with P301L mutation in the forebrain at levels 13-fold higher than endogenous mouse tau (Ramsden et al., 2005). Behavioural tests were conducted from 5.5 months of age and mice sacrificed at 6 months. They found no significant differences in behavioural performance between rTg4510 mice fed SY5 or a control diet. Further, histological and biochemical analyses found no significant changes in tau accumulation following curcumin treatment. Given that SY5 was first designed as a fluorine-19 magnetic resonance imaging probe to detect amyloid deposition, it’s effectiveness against tau accumulation may not be optimal (Yanagisawa et al., 2011).

Okuda et al. (2017) designed, synthesized and evaluated the efficacy of the curcumin derivative, PE859, a dual inhibitor of tau and Aβ aggregation, in a senescence-accelerated mouse prone 8 (SAMP8) model. In this 9-weeks study, SAMP8 mice were given oral administrations via gastric tube of PE859 at three concentrations: 0, 1 mg/k/day, 3 mg/kg/day, from 2 to 4 months of age. They reported a significant improvement in cognitive deficits in spatial working memory (i.e., using Y-maze tests), but not for any other behavioural measure. They did however note that PE859 reduced the amount of aggregated tau and Aβ1-40 in SAMP8 mice. It is possible that a more robust behavioural response would have been noted had curcumin treatment been extended.

## Clinical Studies Examining Curcumin and Tau Pathology

Conclusions on the efficacy of curcumin and its derivatives has been hindered by the limited number of clinical trials, and inconsistent reports on the effectiveness of these compounds in improving cognitive deficits (see Voulgaropoulou et al., 2019). The studies that have directly examined tau pathology in clinical studies have also reported mixed results. Ringman et al. (Ringman et al., 2012) evaluated the efficacy of the Curcumin 3 Complex^®^ (Sabinsa Corporation, Piscataway, NJ), a curcuminoid mixture of curcumin, bisdemethoxycurcumin and demethoxycurcumin, in a population with mild to moderate AD. The compound was administered in three doses (placebo, 2 g/day and 4 g/day curcumin) for 24 weeks. However, Curcumin C3 Complex^®^ did not improve cognitive deficits or reduce tau and Aβ levels in plasma and cerebrospinal fluid (CSF). In this study, low bioavailability of the compound was noted in plasma and patient dropout due to gastrointestinal complains was reported.

In contrast, Small et al. (Small et al., 2018) evaluated the effects of Theracurmin^®^, a form of curcumin with increased intestinal endothelium penetrability, and reported positive effects. In this study, non-demented adults between 51–84 years of age were given Theracurmin^®^ or placebo twice a day for 18 months. The authors found that Theracurmin^®^ improved verbal and visual memory and attention. Further, 2-(1-ethylidene) malononitrile positron emission tomography (FDDNP-PET) scans performed pre- and post-treatment found a reduction in tau and amyloid accumulation in the amygdala of the curcumin treated group compared to placebo. In the hypothalamus, the curcumin treated group showed no change, while the placebo group showed an increase. Together, this indicates that curcumin can reduce pathogenic protein accumulation in the brain. The success of this latter study may be due to 1) it’s length, indicating that extended curcumin usage may be key for improved cognitive function, and 2) it’s use of non-demented adults, suggesting a more preventative than curative role for curcumin in allaying cognitive decline in human populations. For a stronger grasp on curcumin’s effectiveness in treating AD, more clinical studies are necessary, especially in more severely affected populations.

## Future Directions

The general success of curcumin and curcumin derivatives in ameliorating AD pathology in animal studies is encouraging. Although, there has been limited success in clinical trials this may simply be attributable to the small number of such studies having been attempted and published to date. Given that curcumin provides a cost-effective, easily accessible solution to promote resilience to dementia, and that such a holistic treatment for AD will have even greater significance for developing countries, there is an urgent need to level the gap between animal and clinical studies. First, more clinical trials are required, especially with a focus on understanding the ability of curcumin to ameliorate cognitive deficits in severely affected populations and on the benefits of extended curcumin treatment. Other avenues for improvement include finding ways to increase the bioavailability of curcumin in the brain and considering dose dependent effects of curcumin in clinical studies (Voulgaropoulou et al., 2019).

Yet another approach of placing greater emphasis on tau-focused development of curcumin derivatives and subsequent validation in animal models may provide a more fruitful strategy. For example, a number of *in vitro* studies have demonstrated the effectiveness of curcumin derivatives in attenuating the formation of toxic tau oligomers, including tau-targeting biomimetic nanoparticles that reduce phospho-tau levels (Gao et al., 2020) and sugar-curcumin conjugates that suppress tau aggregation more efficiently at lower concentrations than curcumin (Dolai et al., 2011). A study by Cascio et al. (Lo Cascio et al., 2019) screened a small library of novel curcumin derivatives against pre-formed tau oligomers and identified six compounds that interacted with toxic tau species to further promote aggregation. Interestingly, these larger tau structures had decreased toxicity in human neuroblastoma SH-SY5Y cell lines and cultured primary cortical neurons. A subsequent paper examining these compounds and disease-relevant brain-derived tau oligomers (BDTOs) isolated from brain tissues of different tauopathies reported similar results but identified compound CL3 as effective in the formation of larger, less toxic tau aggregates with decreased seeding propensity (Lo Cascio et al., 2020). A thorough validation in animal and clinical studies will reveal if any of these tau-directed curcumin derivatives are more effective in reducing cognitive deficits caused by AD. In summary, the work summarised here highlights a great potential for curcumin as a therapeutic treatment for AD. With a continued push towards development and testing of more effective curcumin derivatives, future studies may finally bridge the gap in translation.
